# Case report: Off-label use of low-dose perampanel in a 25-month-old girl with a pathogenic SYNGAP1 variant

**DOI:** 10.3389/fneur.2023.1221161

**Published:** 2023-08-17

**Authors:** Siddharth Gupta, Yun Hwang, Natasha Ludwig, Julia Henry, Shilpa D. Kadam

**Affiliations:** ^1^Department of Neurology, Kennedy Krieger Institute, Johns Hopkins University School of Medicine, Baltimore, MD, United States; ^2^Neuroscience Laboratory, Hugo Moser Research Institute at Kennedy Krieger, Baltimore, MD, United States; ^3^Department of Neuropsychology, Kennedy Krieger Institute, Johns Hopkins University School of Medicine, Baltimore, MD, United States; ^4^Department of Psychiatry and Behavior Psychology, Johns Hopkins University School of Medicine, Baltimore, MD, United States; ^5^Department of Pediatrics, University of Chicago, Chicago, IL, United States

**Keywords:** neurodevelopmental disorder, seizures, anti-seizure medication, perampanel, SYNGAP1

## Abstract

**Introduction:**

Preclinical studies in a mouse model have shown that SYNGAP1 haploinsufficiency results in an epilepsy phenotype with excessive GluA2-AMPA insertion specifically on the soma of fast-spiking parvalbumin-positive interneurons associated with significant dysfunction of cortical gamma homeostasis that was rescued by perampanel (PER), an AMPA receptor blocker. In this single case, we aimed to investigate the presence of dysregulated cortical gamma in a toddler with a pathogenic SYNGAP1 variant and report on the effect of low-dose PER on electroencephalogram (EEG) and clinical profile.

**Methods:**

Clinical data from physician's clinic notes; genetic testing reports; developmental scores from occupational therapy, physical therapy, speech and language therapy evaluations; and applied behavioral analysis reports were reviewed. Developmental assessments and EEG analysis were done pre- and post-PER.

**Results:**

Clinically, the patient showed improvements in the developmental profile and sleep quality post-PER. EEG spectral power analysis in our patient revealed a loss of gamma power modulation with behavioral-state transitions similar to what was observed in Syngap1^+/−^ mice. Furthermore, the administration of low-dose PER rescued the dysfunctional cortical gamma homeostasis, similar to the preclinical study. However, as in the epileptic mice, PER did not curb epileptiform discharges or clinical seizures.

**Conclusion:**

Similar to the Syngap1^+/−^ mice, cortical gamma homeostasis was dysregulated in the patient. This dysfunction was rescued by PER. These encouraging results necessitate further validation of gamma dysregulation as a potential translational EEG biomarker in SYNAP1-DEE. Low-dose PER can be explored as a therapeutic option through clinical trials.

## Introduction

SYNGAP1-developmental and epileptic encephalopathy (SYNGAP1-DEE) has been described as a distinct disorder characterized by global developmental delay, moderate-to-severe cognitive impairment, autism spectrum disorder, and generalized epilepsy with spontaneous and reflex seizures (1–3). SYNGAP1 gene (synaptic Ras GTPase-activating protein 1) plays an important role in regulating synaptic function by modulating AMPAR (α-amino-3-hydroxy-5-methyl-4-isoxazolepropionic acid receptor) insertion at post-synaptic membranes (4). We have previously shown that SYNGAP1 haploinsufficiency in a mouse model results in an epilepsy phenotype associated with significant sleep-cycle impairments, excessive GluA2-AMPA insertion specifically on the soma of fast-spiking parvalbumin-positive interneurons (PV+IN) associated with significant dysfunction of cortical gamma homeostasis during behavioral transition states determined using quantitative algorithms run on 24-h electroencephalogram (EEG) recordings (5). AMPAR kinetics in PV+ interneurons are known to control cortical gamma homeostasis associated with cognition, behavior, and sleep (6). AMPAR levels at the synapse are high during wakefulness and low during sleep (7). In Syngap1^+/−^ mice, the haploinsufficiency results in the inability of Ras and Rap proteins to regulate the insertion of AMPAR in the post-synaptic membrane, which results in chronic overexpression of AMPAR (8). Perampanel (PER), which is an AMPAR antagonist, at low doses acutely and significantly rescued the cortical gamma dysfunction on EEG in the mouse model (5).

PER is currently approved for focal onset seizures with or without evolution to the bilateral tonic–clonic seizures in children ≥4 years of age and as an adjunctive treatment of primary generalized tonic–clonic seizures in children ≥12 years of age. The recommended daily maintenance dose range is 8–12 mg daily. These published findings and a webinar talk about the results to a SYNGAP1 family foundation group resulted in a family, who with consultation with their child's physician trialed their 25-month-old daughter with a pathogenic SYNGAP1 variant on low doses of PER. Almost 50% of SYNGAP1-DEE-related epilepsies are refractory to treatment from the very onset, and currently, very few children are prescribed PER even for seizure control such that only 1 of 57 patients in a cohort study was documented to have been on it (3).

In this single case report, we aim to discuss the EEG characteristics including quantitative EEG analysis done in our patient, especially evaluating for the presence of dysfunctional cortical gamma observed and reported in the preclinical EEG studies. Additionally, we also discuss the safety profile, changes in the EEG features, and clinical features, particularly in the developmental profile and sleep pre- and post-PER.

## Methods

The family gave consent to participate in the study for the *post-hoc* analyses of overnight EEGs recorded in their child with identified pathogenic SYNGAP1 variants. Based on the results of the preclinical EEG biomarker study, a family under the guidance of a neurologist initiated an off-label trial of low-dose PER in the patient. The patient was followed up with the neurologist periodically to monitor for side effects and treatment effectiveness. Clinical data from physician's clinic notes; genetic testing reports; developmental scores from occupational therapy, physical therapy, and speech and language therapy evaluations; and applied behavioral analysis (ABA) reports were reviewed.

**Figure 1:**
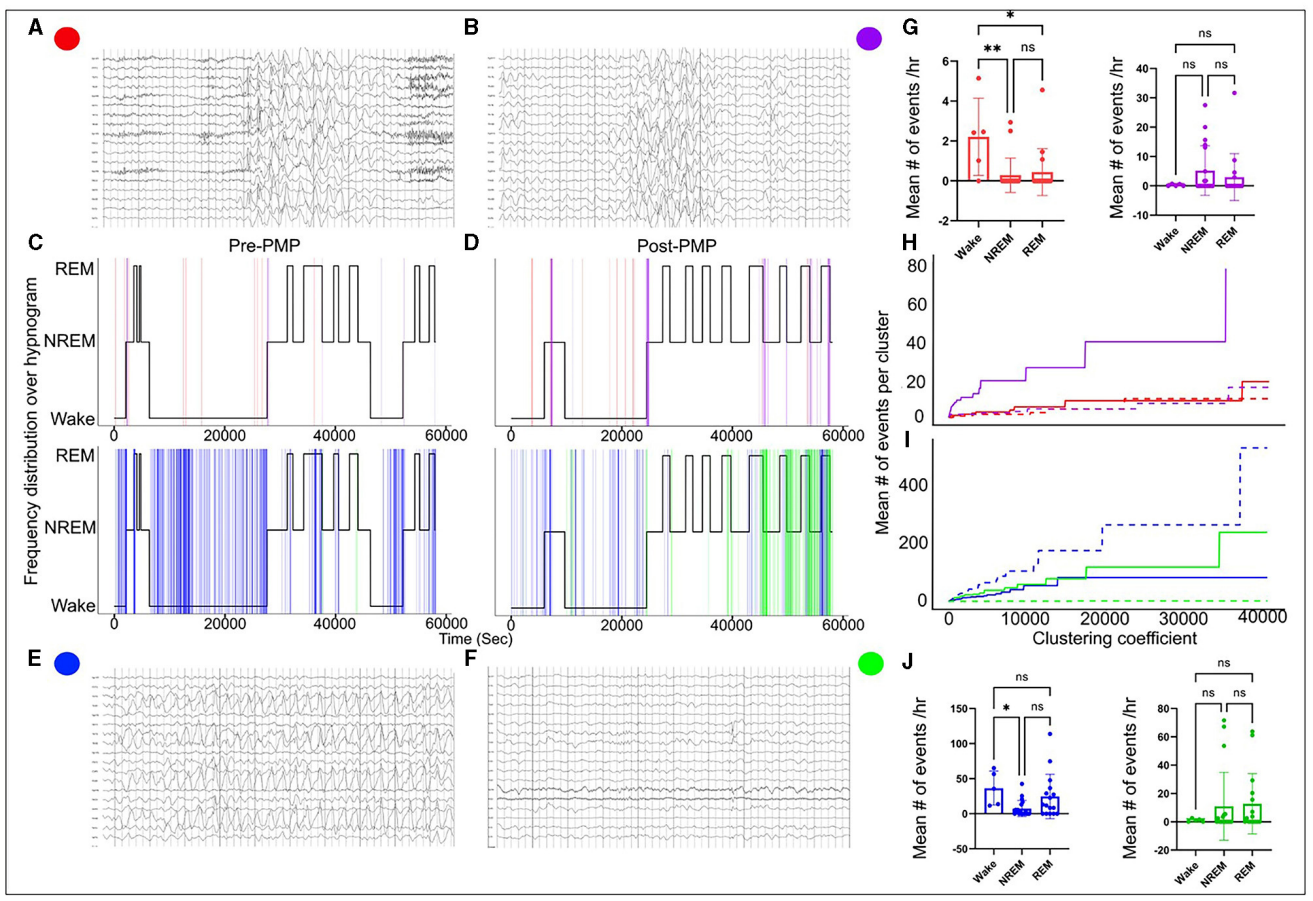
EEG abnormalities pre- and post-PER superimposed over hypnogram. High-amplitude generalized 3–3.5 Hz spike and wave discharges **(A)** and high-amplitude generalized delta activity without spikes **(B)** indicated by red and purple vertical lines on the hypnogram, respectively **(C, D)**. The generalized discharges clustered at the transition from wake to sleep and from REM-to-NREM sleep. Occipital rhythmic delta activity [OIRDA] **(E)** and focal epileptiform discharges over central and temporal head regions over the right **(F)** indicated by blue and green vertical lines on the hypnogram, respectively **(C, D)**. OIRDA was more prevalent in the wake period and REM sleep. It was also significantly reduced post-PER. Focal epileptiform discharges were more prevalent post-PER compared to pre-PER. Abnormal events tended to cluster at state transition points. **(G–J)** Progression of the mean number of events per cluster when the clustering coefficient increased (the dotted line represents pre-PER and the solid line represents post-PER). The clustering coefficient determines the range of a cluster. Although there were differences in progression after the PER administration, no specific patterns were identified. ^*^means *p* < or = to 0.05 and ^**^means *p* < or = 0.01.

EEGs used for analysis were recorded with a 10–20 electrode placement system acquired at a sampling frequency of 512 Hz. EEGs were reviewed in Persyst (version 14, Persyst Development Corporation, Prescott, AZ). Long-duration continuous EEGs (≥16 h) that included awake and sleep states were manually reviewed in non-overlapping 10-s epochs. Each epoch was analyzed for the presence or absence of abnormalities and was given a binary score (1 = present, 0 = absent). The findings that were scored included abnormal rhythmic slowing (generalized or focal, Figures 1A, B) and epileptiform discharges (generalized or focal, Figures 1C, D). Additional analyses were done as follows.

In order to characterize and quantify the clustered nature of different abnormal EEG events (rhythmic slow activity and epileptiform activity), hierarchical clustering analysis was implemented. Hierarchical clustering analysis groups data points based on Euclidean distance and the size of clusters were determined by the clustering coefficient. The average number of specific abnormal EEG events in a cluster was computed for a sequence of increasing clustering coefficients, and the progressions for subtypes of EEG abnormalities were compared. The rate at which the average events increased with respect to the clustering coefficient and size characterized the clustered nature of each abnormal event.

Sleep stages were manually scored in 10-s epochs, and a hypnogram was created. Finally, quantitative EEG analysis was performed. Fast Fourier Transform (FFT) was applied to 10-s epochs in Sirenia Sleep software (Pinnacle Tech. Inc., Kansas USA) for further EEG analysis. Previous reports suggest that Syngap1^+/−^ mice displayed abnormal gamma trends during behavioral transitions between wake and sleep (5). To analyze the presence of similar gamma dysregulation in REM and NREM transition points, linear regression analysis was done on 10 min segment of the REM-to-NREM transition point.

## Results

### Clinical history and EEG pre-PER

The patient was born at 37 weeks gestation to non-consanguineous parents of Chinese descent, the mother aged 34 years and the father aged 38 years. She was born by normal spontaneous vaginal delivery after an uncomplicated pregnancy. Her birth weight was 3.19 kg, and her head circumference was 33 cm. There were no complications immediately after delivery; however, by 7 months of age, developmental delays across several domains became apparent and she began physical, occupational, and developmental therapies through an early intervention program. There were no other significant medical concerns reported by the family.

On physical examination at 18 months of age, by a pediatric neurologist, there were no dysmorphic features identified; however, marked axial and appendicular hypotonia with diffusely diminished deep tendon reflexes were noted. Parents also reported significant social and communication deficits. She would not initiate peer interactions and preferred playing alone. Moreover, many times, she did not play with toys functionally. She also displayed repetitive behaviors such as head banging on the floor. She was eventually formally diagnosed with autism spectrum disorder by a developmental pediatrician. The parents also reported significant sleep dysfunction with multiple nighttime awakenings. There were no other comorbidities reported. There are numerous causes for global developmental delay and autism which includes several genetic, metabolic, and environmental factors. As a part of the etiologic workup, at ~19 months of age, the patient underwent genetic testing with whole-exome sequencing, which revealed a pathogenic variant in the SYNGAP1 gene (c.1167delA,p.G391AfsX12, heterozygous).

**Figure 2:**
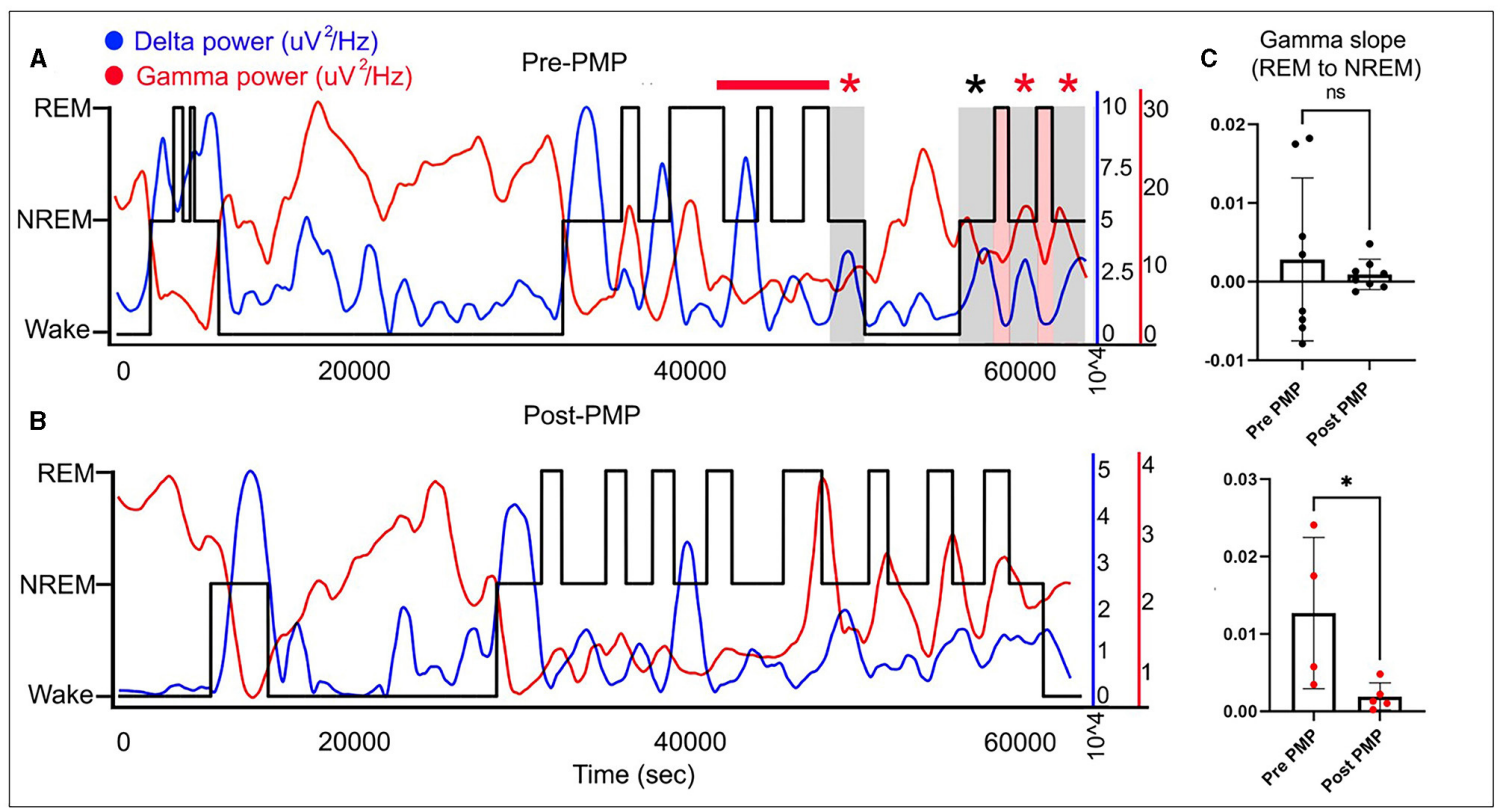
Wake-NREM-REM sleep delta and gamma powers plotted for 16 h EEG both pre- and post-PER over hypnograms (black line) for the same EEG show opposite trends with delta (blue line) going up in NREM slow-wave-sleep and gamma (red line) increasing in wake and REM sleep stages **(A, B)**. Quantification of gamma slopes for behavioral-state transitions from REM-to-NREM shows abnormally increased gamma in the pre-PER EEG that is significantly rescued on the post-PER EEG in the same patient **(C)**. ^*^means *p* < or = to 0.05 and “ns” means *p* > 0.05 (statistically not significant).

The diagnosis of SYNGAP1-DEE prompted the EEG since a majority of patients with SYNGAP1-DEE have epilepsy (3). An overnight continuous 16-h EEG done at 19 months of age showed occasional bursts of high voltage 2.5–3 Hz generalized spike-and-slow-wave activity with an occipital predominance (O1, O2 > P7, P8), particularly in the transition zones between awake to sleep; additionally, seen during awake-to-sleep transitions were high voltage semirhythmic to rhythmic 2–3 Hz delta activity lasting ~2 s; finally, there were frequent runs of medium-to-high voltage 2.5–3.5 Hz notched rhythmic delta activity without evolution or clinical correlation, consistent with occipital intermittent rhythmic delta activity (OIRDA) which at times exhibited fixation-off phenomenon. Finally, rare focal epileptiform discharges over the temporal and central head regions were seen bilaterally (Figure 1). EEG spectral power analysis revealed that similar to dysregulated gamma seen during behavioral transition points from wake to NREM sleep in Syngap1^+/−^ mice, we also observed gamma dysregulation in our patient's EEG, where gamma power remained increased during NREM when transitioning from REM, therefore, presenting a positive slope (Figure 2). Of note, the patient had no clinical seizures.

At 21 months of age, the patient was evaluated by an applied behavior analysis (ABA) agency as part of starting treatment. The evaluation included the Verbal Behavioral Milestones Assessment and Placement Program [VB-MAPP; (9)], which is a criterion-referenced assessment designed to assess verbal and other related social skills typically acquired between 0 and 48 months of age. At this time, the patient demonstrated 7 of the 170 skills/milestones on the VB-MAPP. She was subsequently evaluated at 24 months of age by her early intervention program using the Hawaii Early Learning Profile (HELP), a criterion-referenced measure of developmental skills, and selected subtests from the Peabody Developmental Motor Scale-II (PMDS-II), a standardized and norm-referenced measure of gross motor functioning. The results on domains of the HELP domains were as follows (in age-equivalent months): cognitive = 18, receptive communication = 12, expressive communication = 10, gross motor = 10, fine motor = 18, social and emotional =12, and adaptive = 12. The results on subtests of the PDMS-II were as follows (in age-equivalent months): stationary (i.e., ability to sustain control of her body within its center of gravity and retain equilibrium) = 14 (16th percentile); locomotion (i.e., her ability to transport her body from one base of support to another) = 12 (<1st percentile); and object manipulation (i.e., ability to throw, catch, and kick a ball) = 12 (1st percentile).

### Clinical and EEG changes post-PER

Low-dose PER at 0.2 mg (0.02 mg/kg/day) every night was started when she was 25 months of age, while the patient was still anti-seizure medication treatment naive. The dose was subsequently increased to 0.3 mg every night.

A follow-up overnight EEG was done at 29 months of age which when compared to her prior EEG at 19 months showed several differences. EEG abnormalities pre- and post-PER superimposed over the hypnogram are shown in Figure 1. These abnormalities clustered at different state transitions (wake, REM, and NREM sleep). There was an overall decrease in the prevalence of OIRDA post-PER. Additionally, the EEG evolved from initially demonstrating generalized epileptiform discharges and only rare focal epileptiform discharges to consisting of generalized and frequent multifocal epileptiform discharges in follow-up EEG (Figure 1). Moreover, spectral power analysis revealed that the dysregulated gamma power seen during REM-to-NREM sleep transition points was significantly alleviated after the PER (Figure 2). Her parents first noted clinical seizures when she was 22 months of age which were described as episodes of eye blinking (eyelid myoclonia) that occurred daily. The patient subsequently also developed atonic seizures with “head drops” at ~38 months of age. By 6 months post-initiation of treatment with once daily low-dose PER bedtime, she showed significant improvements in her sleep patterns and developmental skills. Specifically, her parents reported improvement in sleep quality with fewer nighttime awakenings. Early intervention evaluations between 32 and 33 months of age indicated that she demonstrated began walking independently, learned to catch a beach-sized ball, and started to use a pincer grasp. She also demonstrated improved feeding skills including an increased repertoire of preferred foods, improved oral motor development, increased cup and straw cup drinking, and increased independence with utilizing utensils to self-feed. She began using a Picture Exchange System of Communication (PECS) functionally to communicate and was using more approximated vowel shapes and experimenting with some consonants (/h/,/b/) to primarily label items or actions. She also was demonstrating better sustained attention and play skills (i.e., began functional play skills and pretend play). Repeat assessment on the VB-MAPP indicated skill development across time (each point is a skill/milestone): a score of 7 at the time of her 21-month evaluation, 10 at the time of her 28-month re-evaluation, and 17.5 at her 34-month re-evaluation (Table 1).

**Table 1:** VB-MAPP assessment for verbal and social skills (↑, improvement in skill from prior assessment; ↓, worsening in skill from prior assessment; NA, not assessed; PECS, Picture Exchange Communication System).

**VB-MAPP domain**	**3 months post-PER (dose 0.2 mg)**	**10 months post-PER (dose 0.3 mg)**
**Communication**		
Manding (i.e., requesting)	↑	↓
Pointing/gesturing to preferred items	↑	↑
Listener responding- reinforcing/preferred items	↑	↓
PECS-Phase 1	↑	↑
**Cognitive**		
Self-help	NA	NA
Motor imitation	↑	↑
Receptive commands	↑	↑
**Social**		
Responds to name	↑	↑
Parallel play	↑	↑
Independent play	↑	↑
**Health**		
Feeding	↑	↑
**Behavior**		
Functional communication training	↑	↑
Waiting	↑	↑

## Discussion

The patient presented in the case report had several clinical features similar to those described in a large series of patients diagnosed with SYNGAP1-DEE which include developmental delay, autism spectrum disorder, sleep dysfunction, and generalized epilepsy with atonic seizures and eye lid myoclonias (3). There is currently no specific treatment available for patients with SYNGAP1-DEE. The developmental improvements observed after initiation of low-dose PER are promising in this single case; however, the absence of natural history studies in SYNGAP1-DEE makes it challenging to determine whether her developmental improvements on PER are above and beyond what would be expected in patients with SYNGAP1-DEE receiving developmental therapies. It is important to note that the patient was receiving physical therapy 2x/week, speech therapy 2x/week, occupational therapy 2x/week, and ABA therapy during the PER treatment period. This could be further explored in blinded placebo-controlled clinical trials. Low-dose PER was ineffective in controlling clinical seizures similar to preclinical reporting. The seizure types in our patient included eyelid myoclonia and atonic head drops which have been described in prior SYNGAP1 series. The most common type of seizures in SYNGAP1-DEE is generalized seizures including myoclonic, atonic, and myoclonic-atonic seizures; atypical absences; and eyelid myoclonia and myoclonic absences (1, 3, 10–12). Photosensitivity, eye-closure sensitivity, and fixation-off sensitivity (FOS) are reported in some individuals with SYNGAP1-DEE. Other seizure triggers including eating, sounds, and touch have also been described (3, 11–14). The dose of PER in our patient was gradually increased to target better seizure control. At the last clinic follow-up, the dose of PER was at 1.5 mg nightly and the addition of another anti-seizure medication was being considered. The patient did not develop any adverse effects to the low-dose PER.

EEG cortical gamma homeostasis plays a significant role in sleep quality, cognition, and learning (6). This cortical gamma homeostasis which was found to be dysregulated in Syngap1^+/−^ mice in turn resulted from abnormal AMPAR kinetics. PER is a pan-AMPAR antagonist (15). PER-treated Syngap1^+/−^ mice showed a significant rescue of gamma dysregulation. Similarly, our patient's dysregulated cortical gamma was rescued with the off-label treatment with low-dose PER. This cross-species dysregulation of cortical gamma holds significant potential for a novel translatable neurophysiological biomarker and warrants further validation in larger human cohorts. In neurodevelopmental disorders, the development of precise disease-modifying agents requires simultaneous identification of objective and quantifiable biomarkers to help stratify the heterogeneous group of patients and further recognize groups with the most favorable response. Such biomarkers can also serve as early indicators of treatment efficacy. Neurophysiologic biomarkers using studies, such as EEG which is a non-invasive and readily available tool hold significant promise, especially given its high temporal resolution (16, 17).

Upon plotting the paroxysms of rhythmic delta activity and epileptiform activity over a hypnogram in our patient, it was noted that all abnormal activities clustered at different transition states such as wake, NREM sleep, and REM sleep (Figure 1). Several abnormal EEG features seen in our patient are also shared by patients from various SYNGAP1 series. Focal epileptiform discharges are typically multifocal, but an occipital predominance has been reported in several patients in different reports (3, 11–14, 18, 19). We found that the generalized epileptiform discharges and generalized rhythmic delta activity clustered at sleep onset. This phenomenon was also described in prior reports (14). Low-dose PER was not effective in reducing the overall burden of interictal epileptiform activity similar to the findings reported in Syngap1^+/−^ mouse model. Although PER is an anti-epileptic medication, it potentially targets the developmental aspect of SYNGAP1-DEE in low and tolerable doses, but control of seizures requires additional anti-seizure medications. Intermittent rhythmic delta activity is another common finding in patients with pathogenic SYNGAP1 variants (13, 18). The delta activity is often seen in the posterior head regions or is generalized with an occipital/posterior predominance. Specifically, OIRDA which is often notched in morphology has been described in prior reports of patients with SYNGAP1 pathogenic variants. In a series of 15 patients described by Lo Barco et al., 14/15 patients had a notched OIRDA that exhibited fixation-off sensitivity and became near continuous while falling asleep and during the initial phases of sleep (13). Similar features were also seen in our patient. OIRDA is a non-specific interictal EEG abnormality associated with both generalized and focal epilepsy (20). An inverse relationship was noted with the prevalence of OIRDA and epileptiform activity pre- and post-PER. The correlation of the EEG abnormalities to each other and their relation to clinical severity, seizures, or PER could also be explored in future studies.

## Conclusion

Mechanistic insights gained from sound preclinical EEG studies led to the first safe and successful trial of low-dose PER in a patient with SYNGAP1-DEE that potentially led to improvements in developmental measures and sleep. Next, dysregulated cortical gamma during behavioral transition states which was present in Syngap1^+/−^ mice was also present in our patient and thus shows promise as a novel translational biomarker. Finally, the strong preclinical data on the rescue of cortical gamma dysregulation with the use of low-dose PER in conjunction with this case report highlight its potential use to target the sleep, cognitive, and behavioral symptoms seen in SYNGAP1-DEE, recognizing that the treatment of associated epilepsy might require additional anti-seizure medications. Further larger studies on the validation of the qEEG biomarker and the use of PER in SYNGAP1-DEE are warranted.

## Patient perspective

The patient's mother's testimonial is as follows:

“*The progress she has made after perampanel was immediate and hard to miss. About a week after she started taking perampanel, she walked independently for the first time. She was able to ride a tricycle, go play in playgrounds by the time she was 3 years old. At age 5, currently, she is able to participate in gymnastic class, swimming, jump on trampoline, ride the scooter, shooting basketball to name a few. She was able to master her pincer grasp within a few months. She is able to zip and unzip zippers, she can open medicine bottles, she can do puzzles and stack blocks with ease, she can isolate her fingers, and make different gestures and signs*.

*She also did not have much language before perampanel. We could not be certain if she had much understanding of the world around her. We are seeing her transitioning out of babyhood into toddlerhood from a helpless baby to an opinionated toddler who wants her independence. By the time she was 3 years old, she knew her letters, color, shapes, animals… improvement in her cognition was obvious. Fast forward to 2 years later, all her teachers, therapists, even developmental pediatrician would describe her as ‘intelligent' in their reports. She also actively participates in school and therapies and is always excited to learn. In terms of speech, her progress has not plateaued. Even though verbal language is still minimal, she is able use functional communications such as signs, gestures, and her AAC communication device. Her nighttime wakings also dropped by the 3rd week. She no longer woke up in the middle of the night*.

*While on perampanel, her seizures have evolved and gotten harder to control as she grew older. She's now on CBD and Depakote besides perampanel, but her development has not slowed down even though seizure have worsened. I do not want to give the impression that my child became normal after perampanel, she still greatly affected by SynGAP, but comparing her pre and post perampanel in every aspect, her improvement is drastic. I firmly believe that perampanel changed her developmental trajectory [sic].”*

## Data availability statement

The original contributions presented in the study are included in the article/supplementary material, further inquiries can be directed to the corresponding author.

## Ethics statement

The studies involving humans were approved by Johns Hopkins Institutional Review Board. The studies were conducted in accordance with the local legislation and institutional requirements. Written informed consent for participation in this study was provided by the participants' legal guardians/next of kin. Written informed consent was obtained from the minor(s)' legal guardian/next of kin for the publication of any potentially identifiable images or data included in this article.

## Author contributions

SG: design of the work, data analysis, and writing the first manuscript draft. YH and NL: data analysis and contributed to drafting the manuscript. JH: data acquisition and critical revision of the content. SK: conception and design of the work, data analysis, and critical review of the manuscript. All authors agree to be accountable for all aspects of the work and provide approval for publication of the content.
